# Cow’s milk consumption in childhood and adolescence: a Q&A expert review of current evidence

**DOI:** 10.3389/fnut.2026.1857069

**Published:** 2026-09-11

**Authors:** Carlos Alberto Nogueira-de-Almeida, Tulio Konstantyner, Luciana Rodrigues Silva, Elza Daniel de Mello, Mauro Fisberg

**Affiliations:** 1 Department of Medicine, Federal University of São Carlos (UFSCar), São Carlos, Brazil; 2 Department of Pediatrics, Federal University of São Paulo (UNIFESP), São Paulo, Brazil; 3 Department of Pediatric Gastroenterology, Federal University of Bahia (UFB), Salvador, Brazil; 4 Department of Pediatrics, Federal University of Rio Grande do Sul (UFRGS), Porto Alegre, Brazil; 5 Center of Excellence in Nutrition and Feeding Difficulties (CENDA), Pensi Institute, São Paulo, Brazil

**Keywords:** adolescent nutrition, infant nutrition, inflammation, milk, pasteurization

## Abstract

Cow’s milk and its derivatives are a food group of recognized nutritional value during childhood and adolescence, and their consumption is endorsed by the main pediatric societies from 1 year of age. However, studies indicate that a significant portion of Brazilian children and adolescents do not meet the consumption recommendations, compromising the intake of essential nutrients. This expert review critically evaluated the scientific evidence on cow’s milk consumption in this age group, in a question-and-answer format, based on a search in the PubMed/MEDLINE, Scopus, Web of Science and ResearchGate databases, with a final sample of 70 publications. The results indicate that regular consumption of cow’s milk is safe and beneficial for healthy children and adolescents, contributing to physical growth, bone health, favorable body composition, and potential improvement in cognitive performance. Contraindications are restricted to specific conditions, such as cow’s milk protein allergy and lactose intolerance. However, some findings–particularly those related to cognitive performance–remain heterogeneous, highlighting the need for further well-designed studies. It is concluded that, in the absence of these conditions, there is no scientific basis for the exclusion of cow’s milk from the diet of this population group, and its consumption should be and its consumption should be encouraged within the context of a balanced and individualized diet.

## 1 Introduction

After the breastfeeding period, cow’s milk and its derivatives are an important food group in nutritional recommendations for children and adolescents, due to their nutritional composition, impacting different stages of growth, bone development and metabolic health. Although the World Health Organization (WHO) indicates that cow’s milk may be used for non-breastfed infants from 6 months of age in specific contexts, particularly when infant formulas are not accessible, its consumption is more consistently recommended from 12 months of age by several international medical societies (1–7). The analysis of milk and dairy product consumption showed an inverse association between the total consumption of this food group and the prevalence of obesity, due to its levels of high-quality proteins, vitamins, and minerals, particularly calcium (8, 9). At the same time, achieving the proper amount of certain nutrients may be more difficult in the dietary absence of dairy (10). Studies show that many children and adolescents do not consume the recommended amount of milk and dairy products, which can compromise the intake of important nutrients at this stage of life. An observational study conducted in Poland found results suggesting that children aged 10–12 years have insufficient consumption of milk and dairy products (11). A population-based study that evaluated the food intake of 228 Brazilian children aged 4–5 years observed that 76% of them did not reach the recommendation of two daily servings of dairy products and 56% consumed less than one serving per day (12). Another survey, involving more than 100,000 Brazilian adolescents, identified inadequate milk consumption in 58.9% of the population studied (13). Most children over 4 years of age and adolescents do not reach adequate calcium intake, which is the main consequence of reducing dairy consumption in this age group (14). In line with this evidence, Del’Arco et al. (15) when evaluating the food consumption of 188 adolescents (15–19.9 years) and 288 young adults (20–24.9 years), identified, in the group of adolescents, inadequate intake of calcium in 97.28% of the participants, magnesium in 93.13% and sodium in 68.51%. Dietary calcium deficiency is also a worldwide public health concern. In the US population, the prevalence of inadequate calcium intake (<EAR) varied by age group, being lower among children aged 2–3 years (20%–30%) and progressively higher in older groups, reaching approximately 50%–70% in individuals aged 9–18 years (16). In the Canadian population, the prevalence of calcium inadequacy significantly increased in children and adolescents from 2004 to 2015 (17). The low consumption of milk and dairy products contributes to inadequate intake of calcium, magnesium, and phosphorus (15).

In recent years, dairy products have attracted renewed consumer interest, driven by the growing demand for traditional and minimally processed foods, together with the increasing recognition of their nutritional value, particularly their high content of protein and micronutrients (9, 18). However, divergent and sometimes contradictory information about its health effects has generated doubts among consumers. The disconnection between the recommendations for the intake and low consumption of cow’s milk and its derivatives by children and adolescents, even considering the benefits of this food group for the health of this population group, raises the importance of debate among experts in the field about concepts and current scientific knowledge so that nutritional prescription in clinical practice is adequate, respecting the individuality of each child.

Despite the growing popularity of plant-based beverages, it is important to highlight that these beverages do not have a nutritional composition equivalent to that of cow’s milk (19). Therefore, they may not adequately meet the requirements for essential nutrients needed for growth and development during childhood and adolescence, such as calcium and high-biological-value proteins (4).

Although some plant-based beverages may be fortified to more closely resemble the nutritional composition of cow’s milk, the bioavailability of these nutrients may differ. Therefore, plant-based beverages are not recommended as exclusive substitutes for cow’s milk in children’s diets, with the exception of fortified soy-based beverages–which, although they may contain a similar amount of protein to cow’s milk, still have a qualitatively inferior protein profile (20)–in specific situations and under professional guidance. Their consumption may be indicated after 12 months of age in cases such as cow’s milk protein allergy or specific dietary practices, such as veganism (19). Recent evidence suggests that replacing cow’s milk with non-fortified plant-based beverages with the aim of ensuring a sustainable diet may negatively impact the intake of essential nutrients in the pediatric population, particularly calcium, iodine, vitamin B12, riboflavin, and high-biological-value proteins (21, 22). It is important to note that breast milk should be offered exclusively to the baby until the sixth month of life and be maintained, plus complementary feeding, until 2 years or more, followed by continued breastfeeding alongside complementary feeding until 2 years of age or beyond (3). The following information, written by means of questions and answers, refers to the consumption of milk for children from 1 year of age (5, 23) and for adolescents. Dairy products were considered only when the studies or recommendations addressed milk and dairy products together, and not when dairy products were evaluated separately. Therefore, this review does not include issues related to the use of infant formulas, dairy compounds, or products formulated with the addition, replacement, or significant modification of the milk matrix.

In this context, the present study aimed to carry out a critical review of cow’s milk consumption in childhood and adolescence, presenting a structured discussion in a question-and-answer format, focusing on the clarification of nutritional, clinical and health aspects relevant to professional clinical practice based on robust and current scientific evidence.

## 2 Materials and methods

The methodological approach consisted of a structured narrative review informed by expert consensus, rather than a formal systematic review with predefined clinical outcomes.

The selection of topics was guided by the most frequent questions observed in the clinical practice of professionals who work with children and adolescents, such as pediatricians and nutritionists, as well as by recurring discussions related to milk consumption identified in the scientific literature and in professional contexts connected to the dairy product value chain. Therefore, this review was structured around questions considered relevant to clinical practice and to current debates on the topic, which may have influenced the scope of the evidence explored.

The aim of this review was not to systematically assess a single predefined clinical outcome, such as bone health or obesity, but rather to address the main questions and controversies surrounding cow’s milk consumption in pediatric clinical practice. Accordingly, the search strategy was intentionally broad, covering nutritional, gastrointestinal, metabolic, behavioral, and public health aspects, as well as situations in which milk may be recommended, adapted, or, in some cases, restricted. This approach enabled the role of milk in the diets of children and adolescents to be contextualized from different clinical and public health perspectives, rather than restricting the analysis to a single predefined outcome.

The questions formulated were submitted to a panel of 6 experts composed of pediatricians with clinical and scientific experience in the areas of child nutrition and adolescence, who were responsible for the search, critical analysis and interpretation of the pertinent scientific literature.

A literature search was conducted in PubMed/MEDLINE, Scopus, Web of Science, and ResearchGate, with no language restrictions. The search strategy included combinations of controlled descriptors and free-text terms related to cow’s milk consumption and its impact on pediatric health, including “cow’s milk,” “dairy consumption,” “children,” “adolescents,” “nutritional status,” “health outcomes,” “cow’s milk protein allergy,” and “lactose intolerance,” combined using Boolean operators.

Studies were considered eligible if they addressed cow’s milk or dairy product consumption in children and/or adolescents and discussed nutritional, gastrointestinal, metabolic, behavioral, or other health-related outcomes relevant to pediatric clinical practice. Articles published from 2007 onward were also considered, with no language restrictions. Eligible publication types included original studies, systematic reviews and meta-analyses, narrative reviews, international guidelines, position papers, legislative documents, and other relevant scientific or technical publications. Publications focused exclusively on infant formulas, experimental animal studies without direct relevance to pediatric nutrition, conference abstracts without full-text availability, and publications not directly related to cow’s milk consumption in children and adolescents were excluded.

The selection of publications was carried out in two stages, consisting of the screening of titles and abstracts, followed by the full reading of the eligible studies. 121 studies made up the final sample. The selected studies were then classified according to the methodological design, considering the information described by the authors themselves.

Regarding the design, 14 systematic reviews and meta-analyses, 30 narrative reviews, 29 observational studies, 19 position papers, 5 legislative documents, 11 population guidelines, 4 clinical trials, 6 books, 2 experimental analysis and 1 market data were included.

A PRISMA-style flow diagram was developed to summarize the process of study identification and selection.


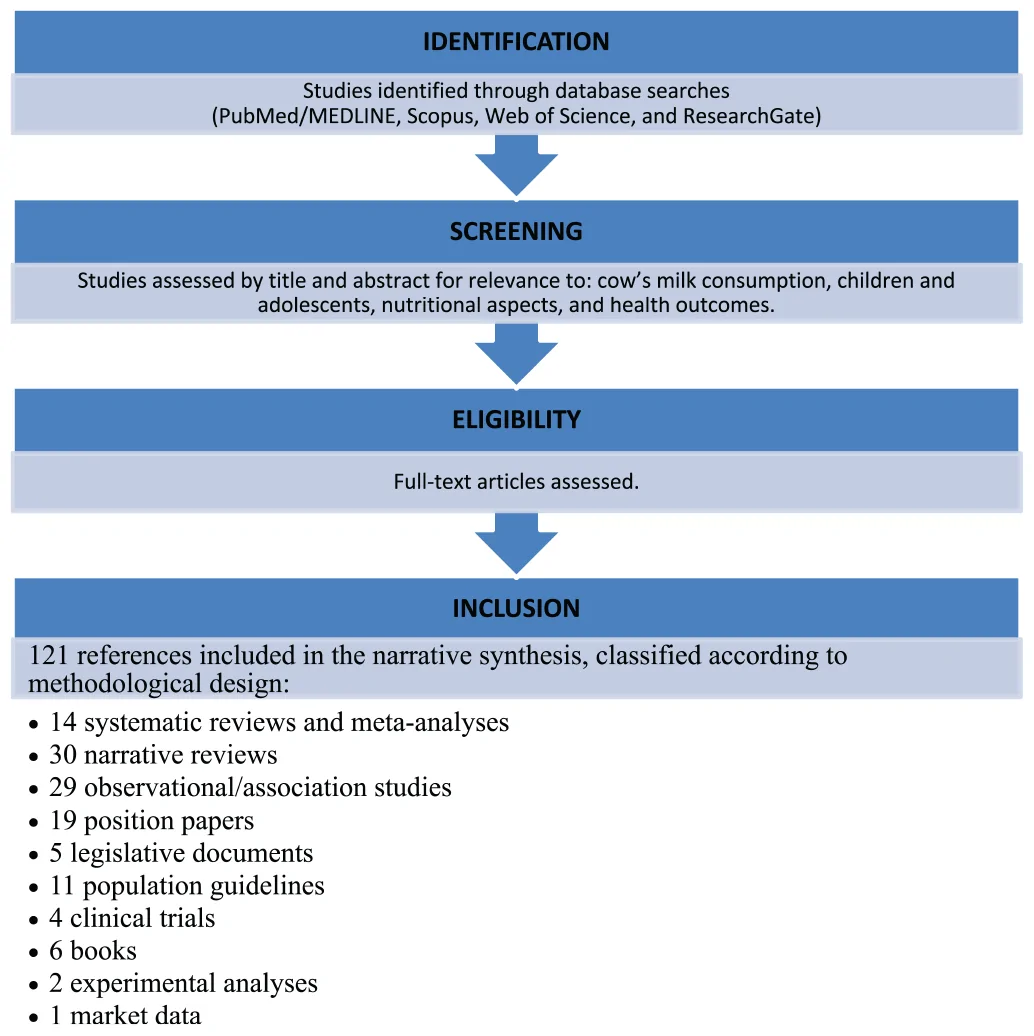


The critical evaluation of the evidence and the elaboration of the text were carried out independently by the experts, with collective discussion and resolution of any disagreements by consensus.

The synthesis of evidence was organized in a narrative manner, prioritizing studies with greater methodological rigor and scientific consistency, being presented in the format of questions and answers.

## 3 Subsections relevant for the subject–Q&A

### 3.1 From what age can cow’s milk be considered in children’s eating plan?

Overall, international societies generally agree in recommending cow’s milk for children from 1 year of age. The World Health Organization (WHO), however, notes that for non-breastfed infants aged 6–11 months, cow’s milk may be used in specific contexts, particularly when infant formulas are not accessible. In such cases, adequate iron intake should be ensured through diet, supplementation, or fortified foods (3).

European Society for Pediatric Gastroenterology, Hepatology and Nutrition (Europe) indicates that cow’s milk may be introduced from 1 year of age, or used in food preparations, such as purées, if used in small amounts. Meanwhile, societies from countries such as Denmark, Canada, Sweden, the United Kingdom, and the United States generally recommend introducing cow’s milk as a beverage around 9–12 months of age or after 1 year of age, while in some cases allowing only small amounts mixed with other foods before that age (5). The Food Guide for Brazilian Children under 2 years of age indicates that, due to different reasons (such as cost, inclusion of new foods and public policies to prevent anemia), from 9 months of age onward, cow’s milk could be part of the infant diet (24). In this scenario, a daily intake of up to 500 mL has been considered safe and compatible with a balanced and healthy diet (25).

According to current recommendations, children from 1 year of age should consume 3 daily servings of dairy products (one serving approx. 180 g) (1–3). ESPGHAN, however, does not establish a specific daily volume to be consumed by this population (5).

For both preschool children (2–6 years old), school aged (7–10 years old) and adolescents (over 10 years old) the milk and dairy products group is described as one of the food groups that should make up a varied and quality diet–with a recommendation for daily consumption up to 600 mL/day (2). Recommendations for milk consumption in early childhood are also supported by a multi-organizational consensus including the American Academy of Pediatrics (AAP), which suggests an intake of 2–3 cups/day of cow’s milk for children aged 12–24 months and up to 2–2.5 cups/day for those aged 2–5 years (26). These recommendations are consistent with AAP clinical guidance emphasizing cow’s milk as a key component of a balanced diet after 12 months of age (27).

It is important to point out that these recommendations do not raise any objection regarding the type of cow’s milk recommended, indicating that there are no restrictions on the form of consumption, including powdered, pasteurized, and UHT milk. The UHT format, for example, is the most widely consumed type of milk in some countries. It is present in approximately 90% of Brazilian households and accounts for 80% of liquid milk sales in Southern European markets, including France, Spain, Italy, and Portugal (28, 29). This is mainly due to practicality, safety, and convenience, since the product has an extended shelf life and does not require refrigeration before opening. These features facilitate availability and storage, transportation, which contributes to longer shelf life and, potentially, fewer losses and lower cost to the consumer (29). Other options, such as powdered milk and pasteurized milk, also have characteristics that favor their adoption by the population according to the profile and reality of each family. Regardless of the format, regular consumption of cow’s milk–in any of its commercial forms–represents a relevant nutritional strategy for children and adolescents, given its profile of nutrients that are important for skeletal growth, muscle development, and the maintenance of physiological functions during childhood and adolescence (3, 10)–benefits that will be explored in the next section.

### 3.2 What is the evidence supporting the health effects of cow’s milk and dairy product consumption in children and adolescents?

Due to its nutritional composition, frequent consumption of cow’s milk has been associated with different health benefits for children and adolescents. Table 1 presents the main data available in scientific articles on different clinical outcomes (bone health, body composition, and cognitive performance).

**TABLE 1:** Effects of milk and dairy consumption on different clinical outcomes.

References	Design	Sample	Clinical outcome
Nutritional adequacy			
Lenighan et al. (12)	Cross-sectional observational study on the impact of dairy product consumption on adequate nutrient intake	228 children between 4 and 5 years old from the *Brazilian Kids Nutrition and Health Survey* (KNHS)	Adding 1 serving of dairy products per day was associated with a 17% reduction in calcium inadequacy (from 86.8% EAR inadequacy to 69.8%), 18% reduction in vitamin A inadequacy (from 48.1% EAR inadequacy to 32.1%), and 3% reduction in zinc inadequacy (from 10.1% EAR inadequacy to 7%).
Souza et al. (37)	Cross-sectional observational study on the prevalence of inadequate micronutrient intake	71.791 adolescents aged 12 to 17 years from the Cardiovascular Risk in Adolescents Study (ERICA)	99% prevalence of inadequate calcium intake, which may be associated with low intake of milk and dairy products
Bone health			
Hidayat et al. (117)	Meta-analysis of randomized controlled trials (parallel or crossover) on the evaluation of milk and dairy consumption on bone health (Nondairy placebo or control or no intervention)	Children and adolescents between 3 and 18 years old	Beneficial effects on bone health [3% increase in mineral content (95% CI; 7.50) and 1.8% (0.016 g/cm2; 95% CI: 0.006, 0.025 g/cm2) increase in bone mineral density and increased height]
de Lamas et al. (118)	Systematic review of 13 studies (controlled trials) on the relationship between dairy intake, height, and bone mineral content	3895 children and adolescents (up to 18 years old)	Dairy consumption is associated with higher bone mineral content (in 6 of 7 studies analyzed) – but not with linear growth in children
Moore et al. (36)	Prospective Cohort Observational Study on the Relationship	106 Children aged 3 to 5 years who have been	Consumption of ≥ 2 servings/day of dairy (compared to a lower intake) was associated with
	Between Childhood Dairy Product Intake and Adolescent Bone Health	followed for 12 years	significantly higher means of bone mineral content (p = 0.009) and bone area (p = 0.019).
Rizzoli (119)	Review of observational studies and randomized controlled trials on the consumption of milk and dairy products and their relationship with bone health in the medium and long term	Children, adolescents, and adults	Avoiding dairy consumption in childhood is a risk factor for fractures: it increases the risk by 4.6x among girls aged 2-20 years Consuming less than 1 serving of dairy per day in childhood increases the risk of fractures by 2x in women aged 50
Herber et al. (116)	Evaluation of a database on milk consumption and risk of underweight and short stature	Children aged 6 to 59 months divided across three different databases containing more than 668.000 individuals.	Milk consumption is associated with a 1.4% lower probability of underweight risk (95% confidence interval −0.02 −0.01) and a 1.9% lower risk of short stature (95% confidence interval −0.02 −0.01).
Zhao et al. (120)	Intervention Study on the Effects of 1-Year Milk Consumption on Bone Mineral Density and Bone Mineral Composition in the Forearm and Calcaneus	215 children aged 4 to 6 years receiving 390ml/d of milk for 12 months compared to 100 children receiving 20-30g of bread	The bread group was used as a control. Compared to this group, children consuming milk had a 7.31% higher BMD and a 7.34% higher BMC in the forearm after 1 year (P < 0.001).
Body composition			
Kang et al. (10)	Meta-analysis on the effects of milk consumption on growth and body composition	2844 Children and adolescents between 6 and 18 years old (17 trials).	Consumption of milk and dairy products for more than 12 weeks was associated with a higher amount of lean body mass (0.21 kg; 95% CI: 0.01) with attenuation in body fat percentage (−0.15 kg; 95% CI: −0.52).
Koca et al. (34)	Cross-sectional observational study on breakfast eating habits and body composition	7116 Children and adolescents between 6 and 18 years old	Milk consumption showed a negative and significant association with BMI z-score (β = −0.103; p < 0.001), indicating that higher milk intake was associated with lower BMI values.
Lu et al. (33)	Systematic review with meta-analysis on the association between dairy consumption and obesity risk	46011 Children and adolescents followed for 3 years (on average)	Children classified in the highest consumption group were 38% less likely to be obese and overweight (pooled odds ratio (OR) = 0.62; 95% confidence interval (CI): 0.49, 0.80). Increasing consumption by 1 serving per day was associated with a 13% reduction in the risk of overweight/obesity (OR = 0.87; 95% CI: 0.74, 0.98).
Wang et al. (35)	Meta-analysis of observational from 33 studies on obesity risk	Children and adults	Children who had the highest consumption of milk and its derivatives had a 46% lower risk of obesity. The evaluation of milk consumption, on the other hand, indicated that children with this habit had a 13% lower chance of obesity.
Cognitive performance			
Zeng et al. (39)	Prospective Cohort Study on Association Between Dairy and Brain Executive Functions	5138 children aged between 6 and 12 years	Children with higher intake have statistically better performance on markers (vs. children with lower intake) such as: task switching (T-score for BRIEF: 46.58 ± 7.48 vs. 45.85 ± 7.10), initiation of activities independently (especially for those with higher milk intake) (T-score for BRIEF 48.02 ± 8.58 vs. 47.14 ± 8.33) and short-term memory (T-score for BRIEF test: 50.69 ± 8.82 vs. 49.89 ± 8.73).
Rumbold et al. (38)	Review on the relationship between milk consumption and cognitive function	Children from 4 to 11 years old	The results are conflicting – however, there is some data showing a positive association of milk consumption with cognitive function, especially among boys who achieve the recommended daily intake of 3-4 servings (3.45% better score on arts and math tests; β: 3.45; 95% CI: 0.67, 6.23)
Inflammation			
Abreu et al. (40)	Cross-sectional study to investigate the association between dairy consumption and metabolic and inflammatory biomarkers.	412 Portuguese adolescents during the years 2011/2012.	Higher levels of total dairy and milk consumption showed an inverse association with IL-6 (P for trend <0.05 for all) in non-obese adolescents.
Aslam et al. (41)	Cross-sectional study to examine the associations between total milk and other dairy consumption and inflammatory markers.	1338 children aged 9 to 13 years.	Milk consumption showed an inverse association with leptin (β: −0.101; 95% CI: −0.177, −0.025; p = 0.009) and a positive association with the adiponectin-leptin ratio (β: …). 0.116; IC 95%: 0.020, 0.211; p = 0.018).
Qureshi et al. (42)	Cross-sectional study on the intake of food groups and C-reactive protein.	4,010 children and adolescents aged 5 to 16 years.	The consumption of milk, fruits, and another specific food subgroup showed a stronger association with lower CRP levels than their respective main food groups.

Milk is considered a nutrient-dense food, providing high-quality protein, calcium, iodine, and B-complex vitamins. Its nutritional composition supports growth, bone health, and metabolism, thereby contributing to adequate nutrient intake in children and adolescents (19, 30, 31).

As shown in Table 1, several recent publications have revealed that the consumption of milk and dairy products may be associated with positive changes in body composition, including increased lean mass and reduced waist circumference and body fat in adults (32), and lower risk of overweight and obesity in children and adolescents (33). This effect was attributed exclusively to milk in the study by Koca et al. (34), which can have a protective action on overweight and obesity when included in breakfast.

These findings, related to body composition (33–35), were endorsed by a meta-analysis of 17 studies: evaluating 2,844 children and adolescents, Canadian researchers demonstrated that the consumption of milk and dairy products was associated with a tendency to acquire a phenotype of better body composition (10).

The consumption of at least two servings of dairy products a day is also associated with higher bone mineral content among adolescents (36). It contributes to the reduction of nutritional inadequacies: the addition of one daily serving of milk (to achieve the recommendation of two daily servings) was associated with a 17% reduction in calcium inadequacy, 18% in vitamin A and 3% in zinc inadequacy (12). In adolescents, a similar scenario is observed considering studies showing that more than 97% of adolescents between 15 and 19.9 years of age have inadequate calcium intake (15). According to Souza et al., this can be attributed, at least in part, to the persistence of the low prevalence of milk and dairy products intake in this age group (37).

In addition to the outcomes described above, recent studies have also explored the association between milk and dairy consumption and cognitive performance in childhood. Observational evidence suggests that higher milk intake may be related to better performance in specific domains of executive functions, although the literature still presents inconsistent results, indicating the need for caution in the interpretation of these findings (38, 39).

Similar to what has been observed in adult populations, the available data indicate that the intake of milk and dairy products is not associated with an increase in inflammatory biomarkers in healthy children and adolescents, and may, in some contexts, even be related to anti-inflammatory profiles.

A cross-sectional study conducted with 412 Portuguese adolescents, the authors found an inverse association between total consumption of dairy products and milk and serum IL-6 concentrations among non-overweight participants (40). Similarly, another cross-sectional study involving 1,338 children aged 9–13 years concluded that milk consumption is associated with a reduction in leptin concentrations in children and positively associated with the adiponectin–leptin ratio, with a statistically significant effect, but of small magnitude and dependent on body fat mass. Leptin can increase the production of inflammatory cytokines, such as CRP, TNF-α, IL-6, and IL-12, and be involved in low-grade inflammation processes. Thus, the inverse association between serum leptin levels and milk consumption suggests anti-inflammatory properties of the food (41).

A cross-sectional study involving 4,010 children and adolescents investigated the association between the intake of different food groups and serum levels of C-reactive protein (CRP), a biomarker of subclinical inflammation. Consumption of cow’s milk and dairy products was not associated with elevated CRP levels. After adjusting for potential confounding factors, including age, sex, race/ethnicity, height, socioeconomic status, and sedentary behavior, it was observed that children in the lowest CRP tercile had higher consumption of grains, vegetables, and dairy products, suggesting an inverse relationship between the intake of these foods and inflammatory markers (42).

Although cow’s milk is recognized as nutritionally important for children and adolescents, excessive consumption, particularly among young children, may be associated with an increased risk of iron deficiency and iron-deficiency anemia. This highlights the importance of a balanced diet and of milk consumption within age-appropriate quantitative recommendations. In addition, some evidence suggests an association between cow’s milk consumption and a higher prevalence of acne during adolescence, possibly related to hormonal modulation and stimulation of the IGF-1 pathway. However, these findings should be interpreted with caution, given the heterogeneity of the studies and the influence of individual factors and overall dietary patterns (43–45).

In summary, the available evidence suggests that the regular consumption of milk and dairy products in childhood and adolescence is associated with nutritional parameters, bone health, body composition and, potentially, cognitive performance, reinforcing the importance of its inclusion in the dietary pattern and its active recommendation by health professionals. It is important to note that the evidence presented is predominantly based on observational studies and therefore reflects associations rather than causal relationships.

### 3.3 Regarding type of cow’s milk (fat content, addition of nutrients and processing), Which is the most suitable for children over 1 year old and adolescents?

#### 3.3.1 Fat content

Considering the lipid profile, milk can be classified as (46):

1. Whole milk: fat content greater than or equal to 3% of its content;
2. Semi-skimmed or partially skimmed milk: intermediate fat content, i.e., 0.6%–2.9% of its content;
3. Skimmed milk: total milk fat content is less than 0.5% of its content.

Several international studies have reviewed milk consumption, including whole milk, in children and adolescents and its effects on weight, growth, and cardiometabolic risk. Overall, they do not indicate a clear health concern associated with whole milk consumption in healthy children; some even suggest a possible association with a lower risk of obesity (47–49).

A 2021 publication showed that among 7,467 children aged 9 months to 8 years, every 1% increase in milk fat content was associated with a 0.05 reduction in BMI z-score–possibly due to impacts on satiety or overall diet composition (50). Data from 2022 corroborates these findings: evaluating the consumption of different types of milk in early childhood, and the researchers demonstrated that the consumption of whole milk (2–3 times/day) was not associated with obesity or cardiometabolic risk during adolescence (51, 52). For children with a clinical diagnosis of obesity or with a family history of dyslipidemia, cardiovascular disease and obesity, the consumption of skim milk can be an alternative to the non-pharmacological therapeutic strategy (46, 51).

Overall, the available evidence in healthy children and adolescents does not indicate that whole milk consumption is associated with adverse effects on obesity, growth, or cardiometabolic risk. On the contrary, studies often report neutral or potentially protective associations with obesity, as well as favorable associations with growth and lean mass. Some guidelines, such as the German guideline on breastfeeding and complementary feeding, caution against a potential increased risk of obesity in adulthood, but only in the context of excessive cow’s milk consumption during childhood (53).

However, as most of the evidence is observational, decisions regarding the type of milk consumed should consider the child’s overall dietary pattern and health status, with individualized guidance provided by a healthcare professional.

#### 3.3.2 Nutrient addition

In addition to the classification according to fat content, still in the field of nutritional aspects, it is important to highlight enriched milks, which can be added with vitamins, minerals, proteins and/or fibers. In the United States, vitamin fortification of fluid milk is optional, as stipulated in the Code of Federal Regulations (21 CFR §131.110). If added, vitamin A must be present at a minimum of 2,000 International Units (IU) per quart (946 mL), and vitamin D at 400 IU per quart, both within limits of good manufacturing practice (54). In Brazil, the supply of these products on the market is regulated by RDC No. 714/2022, which establishes specific criteria for the classification and concentration of added nutrients, and may be an option under special clinical conditions or as recommended by a professional (55).

In addition to naturally providing high-biological-value proteins, calcium, phosphorus, and other micronutrients essential for growth and development, milk may also serve as an important vehicle for nutritional fortification, further enhancing its potential health benefits for children and adolescents. Among fortified micronutrients, vitamin D is particularly relevant because of its role in calcium absorption, bone mineralization, and the prevention of osteometabolic disorders during critical periods of growth. In this context, several countries have adopted vitamin D fortification of milk as a public health strategy, especially in regions with limited sun exposure and a high prevalence of vitamin D insufficiency (56).

Observational studies show that fortified milk consumption contributes significantly to daily vitamin D intake and to improved serum 25-hydroxyvitamin D [25(OH)D] status in the population. In a review conducted by Itkonen et al., it was observed that, in countries with established fortification policies, such as Canada, the United States, and Finland, fortified milk accounted for approximately 28%–63% of the total vitamin D intake of the population assessed. In Finland, for example, after the expansion of national fortification policies in 2003, a substantial improvement in population serum vitamin D levels was observed, with a marked increase in the proportion of individuals with adequate 25(OH)D concentrations (57).

This study compiled data from Irish and British children and adolescents, in countries where vitamin D fortification of milk is not mandatory and found that usual total vitamin D intake ranged from 2.8 to 3.5 μg/day, while dietary intake ranged from 1.6 to 2.6 μg/day. By contrast, in countries with vitamin D fortification policies, namely the United States, Canada, and Finland, mean dietary vitamin D intake among children ranged from 4.4 to 5.9 μg/day, with 2.3–3.3 μg/day derived from dairy products, representing more than half of total dietary intake (57).

#### 3.3.3 Processing

When considering processing, the types of milk available cover different presentations:

1. Refrigerated raw milk: raw milk produced on dairy farms, refrigerated after collection, and intended for milk and dairy product processing establishments subject to official inspection.
2. Pasteurized milk: fluid milk submitted to one of the pasteurization processes provided for in current legislation, automatically bottled in a closed circuit and intended for direct human consumption (54, 58).
3. UHT (Ultra High Temperature) milk: homogenized milk that has been subjected for 2–4 s to a temperature between 130 °C and 150 °C (using a continuous flow thermal process), immediately cooled to a temperature below 32 °C and packaged under aseptic conditions in sterile and hermetically sealed packaging (54, 55, 58).
4. Milk powder: product obtained by dehydration of cow’s milk, whole, skimmed or partially skimmed and suitable for human consumption, by technologically appropriate processes.

Raw milk poses a significant microbiological risk, particularly due to the potential transmission of pathogens such as *Listeria monocytogenes*, *Salmonella* spp., *Campylobacter* spp., *Escherichia coli*, *Brucella* spp., and *Mycobacterium bovis* (58, 59). Accordingly, major public health agencies discourage raw milk consumption, and several countries maintain restrictive regulations on its sale and distribution. According to the FDA, the interstate sale of raw milk has been prohibited since 1987. In the European Union, raw milk may be sold in some countries, but its commercialization is subject to strict sanitary controls, specific labeling requirements, refrigeration, and microbiological monitoring (58, 60).

On the other hand, there are no restrictions on the consumption of milk by children and adolescents as to the form of presentation as a product (liquid or powder) and as to the type of processing (pasteurized milk, UHT or powdered milk), as long as it undergoes adequate heat treatment and follows the sanitary standards recommended.

For the proper preparation of powdered milk, safe drinking water and properly sanitized utensils should be used (61). The product should be reconstituted according to the manufacturer’s instructions, preferably with previously boiled water when recommended. From a public health perspective, special attention should be given to settings with limited access to treated water and sanitation, particularly in developing and underdeveloped countries, where access to safe water and basic sanitation remains a challenge. In these contexts, the use of untreated or contaminated water to prepare milk may substantially increase the risk of gastrointestinal infections and waterborne diseases, especially among children, who are more vulnerable to dehydration and enteric infections. In addition to sanitary concerns, the need for filtered water may also create an additional cost for families, potentially affecting the accessibility and feasibility of regular consumption of this type of product (62).

### 3.4 Is UHT milk safe for consumption by children and adolescents?

Yes, UHT milk, as defined above, is obtained through high-temperature heat treatment followed by cooling and aseptic filling in cartons (63). That is why it is popularly called “carton milk,” being a safe option for children and adolescents, as well as for adults and the elderly.

Heat treatment is essential to ensure the microbiological safety of milk. Regardless of the type of treatment, whether pasteurization or UHT processing, some changes in milk composition may occur, such as protein denaturation. It is worth mentioning that denaturation of milk proteins may be beneficial, as it can improve digestibility and reduce allergenic potential. Regarding micronutrient profile, the main vitamins and minerals in milk, such as calcium, B-complex vitamins, and vitamin D, do not undergo significant changes, ensuring a composition similar to pasteurized milk (59, 64).

Due to its extended shelf life and the fact that it does not require refrigeration before opening, UHT milk represents an important strategy to expand access to dairy products, especially in regions with limited cold-chain infrastructure or logistical challenges in food distribution (65, 66).

The UHT method is approved by international and national health agencies and regulated by Ordinance 370 of September 1997 of MAPA, through the RTIQ–Technical Regulation of Identity and Quality of UHT milk (54, 55, 58, 63). The procedure aims to eliminate and/or inactivate microorganisms that may cause changes in the quality of the product (67).

According to a joint report by the Food and Agriculture Organization of the United Nations (FAO) and the World Health Organization (WHO), UHT processing preserves the nutritional profile of milk, being comparable to pasteurized milk in terms of nutritional value, flavor and color (67), information corroborated by reports from the International Dairy Federation, which highlight that several studies indicate that UHT milk has protein quality equivalent to that of raw milk (68).

A Brazilian study evaluated the effects of pasteurization and UHT processes on the nutritional composition and biochemical parameters of bovine milk. The researchers demonstrated that they maintained the protein and lactose content like raw milk, with a small (but without statistical significance) change in the fatty acid profile–indicating that there are no changes in the nutritional composition, which reinforces its safety for human consumption, including children and adolescents (64). Regarding calcium, the Food and Drug Administration (FDA) points out that both its concentration and bioavailability remain unchanged after pasteurization or ultra-pasteurization (64).

An industrial-scale study evaluating different heat-processing methods found that macronutrients and total mineral concentrations–including calcium, phosphorus, magnesium, potassium, sodium, and zinc–were preserved after UHT treatment. These findings indicate that the overall mineral profile of milk is maintained, while UHT processing also provides important advantages in terms of microbiological safety, stability, and extended shelf life (69).

In the case of “UHT milk,” after heating and cooling, the milk is packaged aseptically in “long-life” carton packages, composed of six layers that protect against light, oxygen, humidity and microorganisms. This system guarantees a longer shelf life for the product without the need for preservatives, whose use, in addition to being dispensable, is prohibited by law in many countries (54, 55, 58, 63, 70). The only substances allowed in this process are stabilizers, which ensure homogeneity and stability of the product during the process and storage (71, 72). The use of these stabilizers is regulated and undergoes rigorous studies and research to ensure their safety (73).

Therefore, aseptic filling and carton packaging use meet the need to extend the shelf life of food by optimizing quality and reducing product cost. A recent narrative review suggests that the use of cartons is considered safe for health, as the different layers of coating ensure that the existing aluminum does not come into direct contact with the food (72). In addition, as previously mentioned, milk with UHT treatment is within the milk options indicated in the Food Guide for the Brazilian Population, being classified as a minimally processed food and considered a safe option with practical advantages, such as: it does not require dilution; can be stored at room temperature while unopened; maintaining its nutritional characteristics (64).

Finally, UHT processing may also be relevant in terms of microbiological safety, stability, and distribution logistics. The possibility of storage at ambient temperature before opening facilitates transportation, availability, and access to milk in regions with limited cold-chain infrastructure, an aspect that is particularly relevant in low- and middle-income countries (74, 75).

### 3.5 When is the consumption of cow’s milk not recommended or recommended with some precautions?

In addition to considerations regarding cow’s milk consumption during the first year of life–a period in which most guidelines and medical societies recommend avoiding or restricting its use, although the WHO allows it as an alternative for non-breastfed infants from 6 months of age (3–6, 19)–some other specific clinical situations may require the total or partial exclusion of cow’s milk for children, adolescents and adults.

#### 3.5.1 Cow’s milk protein allergy (CMPA)

Cow’s milk protein allergy (CMPA) is an immune-mediated reaction to cow’s milk proteins, which can involve multiple organ systems, including the gastrointestinal tract. The IgE-mediated response results in the rapid onset of easily recognizable allergic symptoms, some of which may require emergency treatment. However, delayed (i.e., non-IgE/cell-mediated) or mixed (IgE and cell-mediated) reactions produce a range of symptoms that overlap with other conditions and vary widely in onset and severity (76). Among these are gastrointestinal, respiratory, dermatological manifestations, and there can often be chronic conditions with nutritional impairment. The prevalence of this clinical condition varies by country, age, socioeconomic status, and diagnostic method, such as parental report versus oral food challenge, and depends on whether only IgE-mediated forms or both IgE- and non-IgE-mediated forms are considered. Current data on the prevalence of cow’s milk protein allergy (CMPA) in Brazil, although scarce, indicate low rates: an incidence of 2.2% and a prevalence of 5.4% in children up to 2 years of age (77, 78). In European countries such as the United Kingdom, Italy, Germany, and Greece, prevalence ranges from 0% to 1.3% (79, 80). Latin American studies, including those from Chile and Argentina, and studies from the Middle East report values of approximately 2%–5%, with greater variation according to socioeconomic status within the same country, as observed in Chile: 1.5% versus 9.2% (81–84).

In addition, 87% of them stop having the allergy by the age of 3 and 97% by the age of 15. In a study pointed out by the new guideline of the Brazilian Society of Pediatrics and the Brazilian Association of Allergy and Immunology, although 10.8% of adults (18–65 years old) reported symptoms, only 1% of them were characterized as allergic after medical evaluation (85). Other references confirm the tendency for CMPA to regress with increasing age. In older children and in the adult population, the prevalence of this condition is approximately 0.16%–0.49% (80, 86).

Currently, the treatment recommendation for CMPA is the exclusion of cow’s milk for a period depending on the clinical manifestations, with a provocation test for its proper diagnosis until the remission of symptoms. The pediatrician should pay attention to cases of overdiagnosis or underdiagnosis of this condition and its therapeutic conduct to avoid nutritional problems and predisposition to feeding difficulties (85, 87).

Although about 23% of parents reported food allergies in their infants, in a study published in 2016, only 1.9% were confirmed by oral provocation tests–which indicates the importance of adequate clinical diagnosis to avoid unnecessary exclusions and nutritional restrictions (88).

#### 3.5.2 Lactose intolerance

Although CMPA (cow’s milk protein allergy) and lactose intolerance are the most common adverse reactions to cow’s milk, the pathophysiology of these conditions is completely different. Whereas CMPA is caused by an exaggerated immune response and may lead to severe reactions, lactose intolerance is a consequence of reduced or absent intestinal lactase production, resulting in poor lactose digestion and can determine distension of intestinal loops, diarrhea and abdominal pain. Symptoms occur specifically after ingestion of lactose-containing foods–and are not associated with other systemic reactions (e.g., skin or respiratory symptoms) (89). It can be congenital, primary, or secondary to other clinical conditions, such as malnutrition.

In these cases, the recovery of mucosal integrity can partially or totally restore the production of the enzyme lactase. Patients with primary intolerance, common in the aging process, or with secondary intolerance, usually maintain a residual capacity for lactase production, and it is possible to eat foods with lactose, if this amount does not exceed the individual threshold (90, 91) and lactose-free or “zero lactose” versions of milk and other dairy products may also be incorporated into the diet. It should be noted that a positive genetic test does not mean that the individual has lactose intolerance, only that it will manifest itself at some point in life (91).

It is worth noting that the symptomatology of lactose intolerance is common to several other conditions, which makes diagnosis challenging. The “Consensus of the Brazilian Association of Nutrology and the Brazilian Society of Food and Nutrition on the consumption of cow’s milk by humans” also indicates that the exclusion of milk due to self-perception of intolerance, without confirmation by clinical diagnosis, can result in significant nutritional losses (71). Still according to the European Food Safety Authority (EFSA), it is important to note that there is substantial individual variability in lactose intolerance, and no universal threshold applies to all individuals with this condition. The document further emphasizes that self-reported symptoms do not always correspond to true malabsorption, and that subjective perception alone should not be considered sufficient grounds for the complete exclusion of milk (92).

Among the options available for lactose intolerant patients, lactose-free milks have been shown to be effective in clinical practice, ensuring their contribution to protein and micronutrient intake, since the lactose-free version maintains the nutrients of regular milk (93). In addition, the patient can ingest lactase, which is present on the market, before starting to consume whole milk.

#### 3.5.3 Intestinal discomfort unrelated to lactose intolerance

Functional gastrointestinal disorders (FGIDs), as defined by the Rome IV criteria of the Rome Foundation International Consensus, include conditions such as irritable bowel syndrome (IBS), functional dyspepsia, and functional abdominal pain. These disorders are characterized by chronic gastrointestinal symptoms without evidence of organic or biochemical abnormalities, and lactose intolerance is specifically excluded as the primary cause (94, 95).

These disorders involve visceral hypersensitivity, motor dysfunction, and gut microbiota dysbiosis, with low-grade inflammation and alterations in the gut–brain axis, independently of lactose malabsorption. However, a dietary factor frequently implicated in these conditions is sensitivity to FODMAPs, poorly absorbed fermentable carbohydrates that promote an osmotic effect, excessive colonic fermentation, and gas/hydrogen production, exacerbating symptoms in up to 70% of IBS cases. Therefore, in these cases, the adoption of a low-FODMAP diet is recommended, as it appears to improve symptoms in 50%–75% of patients with IBS by modulating the microbiota, intestinal permeability, and mast cell activation via bacterial LPS, according to international meta-analyses (96–98).

Some gastrointestinal disorders may also be associated with the consumption of A1 beta-casein. Beta-casein is the main protein in cow’s milk, accounting for approximately 30% of its total protein content, and occurs in two main forms: A2, considered the original variant, and A1, which arose from a genetic mutation approximately 5,000–10,000 years ago.

When milk containing A1 beta-casein is digested, intestinal enzymes produce a bioactive peptide called beta-caseomorphin 7 (BMC7), which has opioid properties, binds to intestinal receptors, and can trigger imbalances in intestinal homeostasis, including increased permeability of the intestinal mucosa. On the other hand, A2 beta-casein, when digested, generates minimal amounts of BMC7–which may result in fewer harmful effects. Thus, for cases of intolerance to BMC7, A2 milk has been translated as an alternative strategy, which is currently being studied (89, 99, 100).

In individuals who report gastrointestinal symptoms associated with cow’s milk consumption, in the absence of a confirmed diagnosis of lactose intolerance, evidence suggests that the replacement of A2 milk (which does not contain A1 type casein) may constitute a promising therapeutic alternative and, consequently, remission of intolerance symptoms (89, 99, 100).

It is worth noting that intestinal discomfort and bloating can be common isolated symptoms or be associated with various other conditions such as functional dyspepsia, constipation, and intestinal motility disorders. Among the main causes, gas retention, visceral hypersensitivity, gastrointestinal motility alterations, abdominal wall dysfunction, psychosocial factors, and food intolerances can be mentioned (101, 102). In view of the multiplicity of factors involved in the genesis of these symptoms, the importance of evaluation by a qualified health professional for the correct diagnosis and definition of clinical conduct is highlighted. The exclusion of milk and its derivatives, when performed without formal clinical indication, can result in inadequate nutrient intake. In this context, the National Institutes of Health recognizes dairy self-restriction associated with self-diagnosis of lactose intolerance as a public health problem (71).

### 3.6 Is cow’s milk consumption associated with higher mucus production?

There is a common belief that consuming cow’s milk can increase mucus production in the respiratory tract. This is because it is speculated that beta-casein (BMC) could stimulate mucus production and secretion from MUC5AC glands in the respiratory tract when there is acute inflammation, especially among individuals who have previously had a previous increase in mucus production (103). However, this hypothesis has not been confirmed by most studies, since milk consumption does not seem to exacerbate such symptoms, even in asthmatic patients, indicating that it is not possible to establish a direct relationship (104). Israeli researchers were unable to demonstrate an association between milk consumption and respiratory symptoms when they compared asthmatic children with non-asthmatic children who consumed milk or soy beverage (105).

Thus, considering the current evidence and the nutritional quality of milk and its impacts on children’s health, there is not enough scientific basis to support the exclusion of milk from the diet of children and adolescents, aiming to reduce mucus production or control respiratory symptoms.

### 3.7 Can cow’s milk consumption cause excess protein intake?

Cow’s milk is an important source of quality protein for children and adolescents, except for infants, whose main source is breast milk. Adequate protein intake at all stages of life is essential for maintaining health, and there are recommendations for daily consumption, as shown in Tables 2, 3.

**TABLE 2:** Recommended daily protein intake for children and adolescents.

Age group	Protein RDA (g/day)	% RDA in a glass of milk
1–3	13	49
4–8	19	34
9–13	34	19
14–18	52 (M)/46 (W)	12 (M)/14 (W)

Source: IOM – Institute of Medicine (USA), 2005 (108).

**TABLE 3:** Comparison of different milk intake recommendations.

References	Age	Recommendation	Amount of milk per day
American Academy of Pediatrics (26, 27), Academy of Nutrition and Dietetics, American Academy of Pediatric Dentistry and American Heart Association (19).	0–12 months	Cow’s milk is not recommended	–
	12–24 months	Whole cow’s milk is recommended	2–3 cups (480–720 mL)
	2–3 years	Reduced-fat cow’s milk is recommended	2 cups (480 mL)
	4–5 years	Reduced-fat cow’s milk is recommended	2.5 cups (600 mL)
Dietary Guidelines for Americans (4)	0–12 months	Cow’s milk is not recommended	–
	12–23 months	Whole cow’s milk is recommended	2 cups (400 mL)
	2–8 years	Reduced-fat cow’s milk is recommended	2–2.5 cups (400–600 mL)
	9–13 years	Reduced-fat cow’s milk is recommended	3 cups (720 mL)
European Society for Pediatric Gastroenterology, Hepatology and Nutrition (ESPGHAN)^a^ (5)	0–12 months	Cow’s milk is not recommended	–
	From 12 months	Whole cow’s milk is recommended	–
Brazilian Society of Pediatrics (24).	0–12 months	Cow’s milk is not recommended	–
	From 12 months	Whole cow’s milk is recommended	600 mL/day, equivalent to 3 servings of 200 mL

*^a^*There is no official position from ESPGHAN regarding recommended milk consumption amounts for children and adolescents.

According to the literature, the “Early Protein Hypothesis” suggests that high protein intake during the first 2 years of life–particularly from dairy-based foods–may be associated with greater weight gain, increased IGF-1 levels, and a higher risk of childhood obesity (106, 107). However, it is important to note that this evidence refers to excessive protein intake. When consumed in amounts consistent with medical society recommendations and as part of a healthy dietary pattern, milk should not lead children to exceed the recommended daily intake of this nutrient. In this context, a systematic review and meta-analysis identified an inverse association between dairy product consumption and the prevalence of obesity in children and adolescents (8).

The study of Lenighan et al. that included 228 preschool children (4–5 years) demonstrated that the daily consumption of two servings of milk was safe to recover the inadequacy of micronutrients (calcium, vitamin A and zinc) without resulting in protein excess and health risk (12). Similar results presented a meta-analysis conducted by Kang et al., which indicated positive effects of milk consumption on body composition, with no association with risk of excess protein consumption (10).

Protein intake from cow’s milk is considered safe when total daily protein intake remains within age-specific dietary recommendations and nutritional requirements (108). This is feasible in a balanced diet for children over 1 year old. At these levels, no adverse effects are observed if the daily consumption of cow’s milk remains within the established recommendations (109). In this way, there is no risk of excess protein with the daily consumption of milk by children and adolescents as part of a healthy and balanced diet, respecting the recommended amounts.

In addition, the quality of the protein present in cow’s milk is also worth mentioning. Regarding the protein fraction, 20% corresponds to whey proteins, while 80% corresponds to casein. Regardless of the type, milk proteins, due to their essential amino acid profile, digestibility and bioavailability, are classified as having high biological value by the FAO (Food and Agriculture Organization). According to the Protein Digestibility–Corrected Amino Acid Score indicator (PDCAAS), Milk proteins have a maximum degree of quality (=1), comparable to albumin, the gold standard in this regard (71).

In this context, protein quality may also influence protein intake by modulating satiety and nutritional efficiency (110). High-quality proteins, such as milk proteins, are associated with greater satiety due to slower digestion, the release of hormones such as peptide YY (PYY) and glucagon-like peptide-1 (GLP-1), and the oxidation of excess amino acids. These characteristics may contribute to meeting nutritional requirements without excessive protein intake. In contrast, proteins with lower digestibility or limited essential amino acid content may require higher intake volumes to achieve equivalent nutritional adequacy (111).

### 3.8 Is cow’s milk consumption compatible with a sustainable diet?

Considering the environmental impact of the food production chain and the concerns related to the sustainability of this process, the importance of promoting healthy diets with a low environmental impact has become an increasingly relevant topic in global nutrition policies and dietary recommendations (112).

In this context, the sustainable production of cow’s milk has been increasingly discussed, particularly in low-income countries, given the nutritional importance of this food. Considering that access to milk may vary significantly across populations with different income and education levels, options that ensure safety and greater accessibility are essential–such as UHT milk or even powdered milk, although the latter requires greater caution, as its preparation involves the use of treated water, which is not always available in developing or underdeveloped countries (62).

The literature has been discussing possible strategies to ensure sustainability within the milk production chain. Current evidence suggests that discussions on sustainable diets for the pediatric population should balance environmental aspects with nutritional adequacy, food security, cultural context, accessibility, and long-term health outcomes (113–115).

## 4 Discussion

The scientific evidence presented here suggests that the consumption of cow’s milk and its derivatives by healthy children and adolescents is safe and can be nutritionally beneficial, contributing to adequate physical growth and sustained development of bone mineral density.

In addition, there are findings of potential positive effects on bone health, nutritional adequacy and body composition, reinforcing that cow’s milk is a valuable source of strategic nutrients during childhood and adolescence. Thus, the inclusion of cow’s milk in the diet of healthy children and adolescents represents a safe and effective practice to support physical development, without adverse implications related to protein intake in children over 1 year of age with a nutritionally balanced diet.

In this context, UHT milk and pasteurized milk a safe and nutritionally adequate options since thermal processing preserves the main nutrients of the food, that is, it is not significantly affected by the industrial process. Because it is practical, accessible, and easy to store, UHT milk represents a viable alternative for families, facilitating the regular inclusion of this food in the eating routine of children and adolescents (74, 75).

Thus, cow’s milk, when properly inserted in the diet, remains a safe strategy with high nutritional value for children and adolescents, and its use should be encouraged whenever possible within a qualitatively and quantitatively balanced diet. In addition to its nutritional benefits, there is no scientific reason to exclude cow’s milk from the diet of this population group, except in cases of specific diseases, such as CMPA. In cases of lactose intolerance, the controlled consumption of foods containing lactose is often possible, respecting the individual tolerance threshold.

Thus, although literature points to relevant and consistent benefits associated with milk consumption during childhood and adolescence, it is important to recognize that certain outcomes remain inconclusive. In particular, the effects on cognition, academic performance, and neurocognitive markers are heterogeneous across the available studies, highlighting the need for future investigations with prospective designs and greater methodological standardization.

Since this review was structured around questions considered relevant to clinical practice and to current debates on the topic, some limitations should be considered when interpreting the findings presented.

Given the wide range of questions related to cow’s milk consumption in pediatric clinical practice, a structured narrative review was considered the most appropriate methodological approach. This format enabled the integration of evidence from different study designs, clinical guidelines, epidemiological data, public health perspectives, and a report containing market data, allowing for a comprehensive discussion of the topic.

Although priority was given to studies with greater methodological rigor and scientific consistency, the possibility of selection bias cannot be entirely ruled out. This study should be interpreted considering the support received from the consulting firm SPRIM PRO. To minimize potential biases, all stages related to literature selection, critical appraisal of the evidence, interpretation of the findings, and manuscript preparation were conducted independently by the authors, based on peer-reviewed literature and recognized international guidelines. The sponsor was not involved in the analyses, interpretations, or conclusions presented in this work.

## References

[1] Nogueira-de-Almeida CA Vilanova KCM Perini TM Ribas Filho D. *Alimentação Da Criança Do Zero a Cinco Anos: Manual Do Departamento De Nutrologia Pediátrica.* Catanduva, SP: ABRAN (2024).

[2] SBP. *Manual De Alimentação: Orientações Para Alimentação Do Lactente Ao Adolescente, Na Escola, Na Gestante, Na Prevenção De Doenças E Segurança Alimentar.* São Paulo, SP: Sociedade Brasileira de Pediatria (2018).

[3] WHO. *WHO Guideline for Complementary Feeding of Infants and Young Children 6-23 Months of Age.* Geneva: World Health Organization (2023). 37871145

[4] U.S. Department of Agriculture and U.S. Department of Health and Human Services. *Dietary Guidelines for Americans, 2025-2030.* 10th ed. Washington, DC: U.S. Department of Agriculture and U.S. Department of Health and Human Services (2025).

[5] Fewtrell M Bronsky J Campoy C Domellöf M Embleton N Fidler Mis N et al. Complementary feeding: a position paper by the European Society for Paediatric Gastroenterology, Hepatology, and Nutrition (ESPGHAN) Committee on Nutrition. *J Pediatr Gastroenterol Nutr*. (2017) 64:119–32. 10.1097/MPG.0000000000001454 28027215

[6] American Academy of Pediatrics Committee on Nutrition. Feeding the infant. 8th ed. In: Kleinman RE Greer FR editors. *Pediatric Nutrition.* Itasca (IL): American Academy of Pediatrics (2019). p. 45–112.

[7] American Academy of Pediatrics Institute for Healthy Childhood Weight. *Healthy Beverage Quick Reference Guide.* Itasca (IL): American Academy of Pediatrics (2024).

[8] Babio N Becerra-Tomás N Nishi SK López-González L Paz-Graniel I García-Gavilán J et al. Total dairy consumption in relation to overweight and obesity in children and adolescents: a systematic review and meta-analysis. *Obes Rev*. (2022) 23:e13400. 10.1111/obr.13400 34881504

[9] Marangoni F Pellegrino L Verduci E Ghiselli A Bernabei R Calvani R et al. Cow’s milk consumption and health: a health professional’s guide. *J Am Coll Nutr*. (2019) 38:197–208. 10.1080/07315724.2018.1491016 30247998

[10] Kang K Sotunde OF Weiler HA. Effects of milk and milk-product consumption on growth among children and adolescents aged 6-18 years: a meta-analysis of randomized controlled trials. *Adv Nutr*. (2019) 10:250–61. 10.1093/advances/nmy081 30839054 PMC6416041

[11] Hamulka J Czarniecka-Skubina E Górnicka M Gêbski J Leszczyńska T Gutkowska K. What determinants are related to milk and dairy product consumption frequency among children aged 10-12 years in Poland? Nationwide cross-sectional study. *Nutrients*. (2024) 16:2654. 10.3390/nu16162654 39203791 PMC11357169

[12] Lenighan YM Tassy M Nogueira-de-Almeida CA Offord EA Mak TN. Milk beverages can reduce nutrient inadequacy among Brazilian pre-school children: a dietary modelling study. *BMC Nutr*. (2022) 8:121. 10.1186/s40795-022-00620-w 36316737 PMC9623914

[13] Silva JB Elias BC Mais LA Warkentin S Konstantyner T Oliveira FLC. Factors associated with inadequate milk consumption among adolescents: national school health survey - pense 2012. *Rev Paul Pediatr*. (2019) 38:e2018184. 10.1590/1984-0462/2020/38/2018184 31778414 PMC6909260

[14] Sociedade Brasileira de Pediatria. *Como Otimizar a Ingestão De Cálcio E O Ganho De Massa óssea Em Adolescentes.* São Paulo, SP: Departamento Científico de Adolescência da Sociedade Brasileira de Pediatria (2017).

[15] Del’Arco APWT Previdelli AN Ferrari G Fisberg M. Prevalence of inadequacy and associated indicators with mineral intake in Brazilian adolescents and young adults. *Revista de Nutrição*. (2023) 36:e220123. 10.1590/1678-9865202336e220123

[16] Yu Z Li Y Ba DM Veldheer SJ Sun L Geng T et al. Trends in calcium intake among the US population: results from the NHANES (1999-2018). *Nutrients*. (2024) 16:726. 10.3390/nu16050726 38474853 PMC10934785

[17] Vatanparast H Islam N Patil RP Shafiee M Whiting SJ. Calcium intake from food and supplemental sources decreased in the Canadian population from 2004 to 2015. *J Nutr*. (2020) 150:833–41. 10.1093/jn/nxz318 31891395 PMC7138660

[18] OECD, FAO. *OECD-FAO Agricultural Outlook 2025–2034.* Paris/Rome: OECD Publishing/FAO (2025).

[19] American Academy of Pediatrics, Academy of Nutrition and Dietetics, American Academy of Pediatric Dentistry and American Heart Association. *Consensus Statement. Healthy Beverage Consumption in Early Childhood: Recommendations from Key National Health and Nutrition Organizations.* Durham (NC): Healthy Eating Research (2019).

[20] Brusati M Baroni L Rizzo G Giampieri F Battino M. Plant-based milk alternatives in child nutrition. *Foods*. (2023) 12:1544. 10.3390/foods12071544 37048365 PMC10094203

[21] Kersting M Kalhoff H Zahn K Belgardt AJ Sinningen K Lücke T. Replacing cow’s milk with plant-based drinks: consequences for nutrient intake of young children on a balanced diet in Germany. *J Health Popul Nutr*. (2025) 44:93. 10.1186/s41043-025-00836-z 40155986 PMC11954288

[22] Biscotti P Tucci M Angelino D Vinelli V Pellegrini N Del Bo’ C et al. Effects of replacing cow’s milk with plant-based beverages on potential nutrient intake in sustainable healthy dietary patterns: a case study. *Nutrients*. (2024) 16:3083. 10.3390/nu16183083 39339683 PMC11434795

[23] Greer FR Sicherer SH Burks AW. Effects of early nutritional interventions on the development of atopic disease in infants and children: the role of maternal dietary restriction, breastfeeding, timing of introduction of complementary foods, and hydrolyzed formulas. *Pediatrics*. (2008) 121:183–91. 10.1542/peds.2007-3022 18166574

[24] Brasil. *Diretrizes E Recomendações Do Guia Alimentar Para Crianças Brasileiras Menores De 2 Anos.* Brasilia, DF: Ministério da Saúde (2024).

[25] WHO. *Guiding Principles for Feeding Non-Breastfed Children 6-24 Months of Age.* Geneva: World Health Organization (2005).

[26] Lott M Callahan E Welker Duffy E Story M Daniels S. *Healthy Beverage Consumption in Early Childhood: Recommendations from Key National Health and Nutrition Organizations.* Durham, NC: Healthy Eating Research (2019).

[27] Fuchs GJ Abrams SA Amevor AA. Older infant-young child “formulas”. *Pediatrics*. (2023) 152:e2023064050. 10.1542/peds.2023-064050 37860831

[28] IndexBox. *Europe - Semi-Skimmed Long-Life (UHT) Milk - Market Analysis, Forecast, Size, Trends and Insights.* (2024). Available online at: https://www.indexbox.io/store/europe-semi-skimmed-long-life-uht-milk-market-analysis-forecast-size-trends-and-insights/ (accessed May 14, 2026).

[29] Associação Brasileira do Leite de Vaca (ABLV). *Annual Report.* São Paulo: ABLV (2024).

[30] Zhang X Chen X Xu Y Yang J Du L Li K et al. Milk consumption and multiple health outcomes: umbrella review of systematic reviews and meta-analyses in humans. *Nutr Metab (Lond)*. (2021) 18:7. 10.1186/s12986-020-00527-y 33413488 PMC7789627

[31] Sharifan P Roustaee R Shafiee M Longworth ZL Keshavarz P Davies IG et al. Dairy consumption and risk of cardiovascular and bone health outcomes in adults: an umbrella review and updated meta-analyses. *Nutrients*. (2025) 17:2723. 10.3390/nu17172723 40944114 PMC12430323

[32] Geng T Qi L Huang T. Effects of dairy products consumption on body weight and body composition among adults: an updated meta-analysis of 37 randomized control trials. *Mol Nutr Food Res*. (2018) 62:1700410. 10.1002/mnfr.201700410 29058378

[33] Lu L Xun P Wan Y He K Cai W. Long-term association between dairy consumption and risk of childhood obesity: a systematic review and meta-analysis of prospective cohort studies. *Eur J Clin Nutr*. (2016) 70:414–23. 10.1038/ejcn.2015.226 26862005

[34] Koca T Akcam M Serdaroglu F Dereci S. Breakfast habits, dairy product consumption, physical activity, and their associations with body mass index in children aged 6-18. *Eur J Pediatr*. (2017) 176:1251–7. 10.1007/s00431-017-2976-y 28799014

[35] Wang W Wu Y Zhang D. Association of dairy products consumption with risk of obesity in children and adults: a meta-analysis of mainly cross-sectional studies. *Ann Epidemiol*. (2016) 26:870–82.e2. 10.1016/j.annepidem.2016.09.005 27756684

[36] Moore LL Bradlee ML Gao D Singer MR. Effects of average childhood dairy intake on adolescent bone health. *J Pediatr*. (2008) 153:667–73. 10.1016/j.jpeds.2008.05.016 18701115 PMC4135716

[37] Souza Ade M Barufaldi LA Abreu Gde A Giannini DT de Oliveira CL dos Santos MM et al. ERICA: intake of macro and micronutrients of Brazilian adolescents. *Rev Saude Publica*. (2016) 50:5s. 10.1590/S01518-8787.2016050006698 26910551 PMC4767036

[38] Rumbold P McCullogh N Boldon R Haskell-Ramsay C James L Stevenson E et al. The potential nutrition-, physical- and health-related benefits of cow’s milk for primary-school-aged children. *Nutr Res Rev*. (2022) 35:50–69. 10.1017/S095442242100007X 33902780

[39] Zeng X Cai L Gui Z Shen T Yang W Chen Q et al. Association between dairy intake and executive function in Chinese children aged 6-12 years. *Front Nutr*. (2022) 9:879363. 10.3389/fnut.2022.879363 35898711 PMC9309784

[40] Abreu S Agostinis-Sobrinho C Santos R Moreira C Lopes L Gonçalves C et al. Association of dairy product consumption with metabolic and inflammatory biomarkers in adolescents: a cross-sectional analysis from the LabMed study. *Nutrients*. (2019) 11:2268. 10.3390/nu11102268 31546602 PMC6835390

[41] Aslam H Jacka FN Marx W Karatzi K Mavrogianni C Karaglani E et al. The associations between dairy product consumption and biomarkers of inflammation, adipocytokines, and oxidative stress in children: a cross-sectional study. *Nutrients*. (2020) 12:3055. 10.3390/nu12103055 33036196 PMC7601178

[42] Qureshi MM Singer MR Moore LL. A cross-sectional study of food group intake and C-reactive protein among children. *Nutr Metab (Lond)*. (2009) 6:40. 10.1186/1743-7075-6-40 19822004 PMC2770558

[43] Juhl CR Bergholdt HKM Miller IM Jemec GBE Kanters JK Ellervik C. Dairy intake and acne vulgaris: a systematic review and meta-analysis of 78,529 children, adolescents, and young adults. *Nutrients*. (2018) 10:1049. 10.3390/nu10081049 30096883 PMC6115795

[44] Domellöf M Braegger C Campoy C Colomb V Decsi T Fewtrell M et al. Iron requirements of infants and toddlers. *J Pediatr Gastroenterol Nutr*. (2014) 58:119–29. 10.1097/MPG.0000000000000206 24135983

[45] Ziegler EE. Consumption of cow’s milk as a cause of iron deficiency in infants and toddlers. *Nutr Rev*. (2011) 69:S37–42. 10.1111/j.1753-4887.2011.00431.x 22043881

[46] Pereira PC. Milk nutritional composition and its role in human health. *Nutrition*. (2014) 30:619–27. 10.1016/j.nut.2013.10.011 24800664

[47] Vanderhout SM Aglipay M Torabi N Jüni P da Costa BR Birken CS et al. Whole milk compared with reduced-fat milk and childhood overweight: a systematic review and meta-analysis. *Am J Clin Nutr*. (2020) 111:266–79. 10.1093/ajcn/nqz276 31851302 PMC6997094

[48] White MJ Armstrong SC Kay MC Perrin EM Skinner A. Associations between milk fat content and obesity, 1999 to 2016. *Pediatr Obes*. (2020) 15:e12612. 10.1111/ijpo.12612 31905266

[49] Beck AL Heyman M Chao C Wojcicki J. Full fat milk consumption protects against severe childhood obesity in Latinos. *Prev Med Rep*. (2017) 8:1–5. 10.1016/j.pmedr.2017.07.005 28856083 PMC5552381

[50] Vanderhout SM Keown-Stoneman CDG Birken CS O’Connor DL Thorpe KE Maguire JL. Cow’s milk fat and child adiposity: a prospective cohort study. *Int J Obes (Lond)*. (2021) 45:2623–8. 10.1038/s41366-021-00948-6 34433906

[51] Nogueira-de-Almeida CA de Mello, de Mello PP de Mello PD Zorzo RA Filho DR. Consenso Da Associação Brasileira De Nutrologia Sobre Manejo Da Dislipidemia Secundária à Obesidade Infanto-Juvenil. *Int J Nutrol.* (2020) 10:161–78. 10.1055/s-0040-1705648

[52] McGovern C Rifas-Shiman SL Switkowski KM Woo Baidal JA Lightdale JR Hivert MF et al. Association of cow’s milk intake in early childhood with adiposity and cardiometabolic risk in early adolescence. *Am J Clin Nutr*. (2022) 116:561–71. 10.1093/ajcn/nqac103 35441227 PMC9348987

[53] Prell C Koletzko B. Breastfeeding and complementary feeding. *Dtsch Arztebl Int*. (2016) 113:435–44. 10.3238/arztebl.2016.0435 27397020 PMC4941615

[54] USA. Part 131—milk and cream. In: Regulations CoF editor. *Title 21 chapter I subchapter B part 131: national archives.* Washington, DC: U.S. Government Publishing Office (2026).

[55] ANVISA. *Requisitos Sanitários Para Enriquecimento E Restauração De Alimentos.* Brasilia, DF: (ANVISA) ANdVS (2022).

[56] Pilz S März W Cashman KD Kiely ME Whiting SJ Holick MF et al. Rationale and plan for vitamin D food fortification: a review and guidance paper. *Front Endocrinol (Lausanne)*. (2018) 9:373. 10.3389/fendo.2018.00373 30065699 PMC6056629

[57] Itkonen ST Erkkola M Lamberg-Allardt CJE. Vitamin D fortification of fluid milk products and their contribution to vitamin D intake and vitamin D status in observational studies-a review. *Nutrients*. (2018) 10:1054. 10.3390/nu10081054 30096919 PMC6116165

[58] European Parliament, Council of the European Union,. Regulation (EC) No 853/2004 of the European Parliament and of the Council of 29 April 2004 laying down specific hygiene rules for food of animal origin. *Off J Eur Union.* (2004) L139:55–205.

[59] Food and Agriculture Organization (FAO). *Milk and Dairy Products in Human Nutrition.* Rome: FAO (2013).

[60] U.S. Food and Drug Administration. *Food Safety and Raw Milk.* Silver Spring (MD): FDA (2024).

[61] Brasil. *Ministério da Saúde. Secretaria de Atenção Primaria à Saúde. Departamento de Promoção da Saúde. Guia alimentar para crianças brasileiras menores de 2 anos / Ministério da Saúde, Secretaria de Atenção Primaria à Saúde, Departamento de Promoção da Saúde. – 1. ed. rev. 3. reimpr.* Brasília: Ministério da Saúde (2022). 264 p.

[62] Headey D. Can dairy help solve the malnutrition crisis in developing countries? An economic analysis. *Anim Front*. (2023) 13:7–16. 10.1093/af/vfac083 36845603 PMC9947325

[63] Amancio OMS Paiva S Domence S Marchioni D Ong T Cassani R et al. *A Importância Do Consumo De Leite No Atual Cenário Nutricional Brasileiro.* São Paulo, Sp: Sban (2015). 28 p.

[64] Pestana JM Gennari A Monteiro BW Lehn DN Souza CFV. Effects of pasteurization and ultra-high temperature processes on proximate composition and fatty acid profile in bovine milk. *Am J Food Technol*. (2015) 10:265–72. 10.3923/ajft.2015.265.272

[65] de Souza AB Stephani R Tavares GM. Stability of milk proteins subjected to UHT treatments: challenges and future perspectives. *Crit Rev Food Sci Nutr*. (2024) 64:12352–62. 10.1080/10408398.2023.2250865 37632425

[66] Datta N Deeth HC. UHT and aseptic processing of milk and milk products. 2nd ed. In: Fuquay JW Fox PF McSweeney PLH editors. *Encyclopedia of Dairy Sciences.* London: Academic Press (2011). p. 699–705.

[67] Von Bockelmann B Von Bockelmann IA. *Long-Life Products: Heat-Treated, Aseptically Packed: A Guide to Quality.* Värnamo, Sweden: Fälth & Hässler (1998).

[68] Fox PF. *Heat-Induced Changes in Milk.* Brussels: International Dairy Federation (1995).

[69] Lalwani S Lewerentz F Håkansson A Löfgren R Eriksson J Paulsson M et al. Impact of thermal processing on micronutrients and physical stability of milk and cream at dairy production scale. *Int Dairy J*. (2024) 153:105901. 10.1016/j.idairyj.2024.105901

[70] European Parliament, Council of the European Union. *Regulation (Ec) No 1333/2008 of the European Parliament and of the Council of 16 December 2008 on Food Additives (Text with Eea Relevance). Document 02008R1333-20251109.* Brussels: European Union (2008).

[71] Nogueira-de-Almeida CA Ribas Filho D Soriano EdA Haubert NJBGB Longo S Amancio OMS. Consensus of the Brazilian Association of Nutrology and the Brazilian Society for Food and Nutrition on the consumption of cow’s milk by humans. *Int J Nutrol.* (2024) 17:e24302. 10.54448/ijn24302

[72] Pimentel C Geraldo A. The use of cardboard packaging in the aseptic container of food and beverages: a narrative review. *Higiene Alimentar.* (2024) 38:1. 10.37585/HA2024.01

[73] CODEX. *General Standard for Food Additives Contract No.: CXS 192-1995.* Rome: FAO/WHO (2025).

[74] Sarkar S. UHT processing - best technology for shelf - life extension of M. *Int J Food Sci.* (2020) 9:1–2. 10.19070/2326-3350-200009e

[75] Gedam K Prasad R Vijay V. The study on UHT processing of milk: a versatile option for rural sector. *World J Dairy Food Sci.* (2007) 2:49–53.

[76] Sathya P Fenton TR. Cow’s milk protein allergy in infants and children. *Paediatr Child Health*. (2024) 29:382–96. 10.1093/pch/pxae043 39539784 PMC11557147

[77] Solé D Silva LR Cocco RR Ferreira CT Sarni RO Oliveira LC et al. Consenso Brasileiro Sobre Alergia Alimentar: 2018 - Parte 1 - Etiopatogenia, Clínica E Diagnóstico. Documento Conjunto Elaborado Pela Sociedade Brasileira De Pediatria E Associação Brasileira De Alergia E Imunologia. *BJAI.* (2018) 2:7–38. 10.5935/2526-5393.20180004

[78] Vieira MC Morais MB Spolidoro JV Toporovski MS Cardoso AL Araujo GT et al. A survey on clinical presentation and nutritional status of infants with suspected cow’ milk allergy. *BMC Pediatr*. (2010) 10:25. 10.1186/1471-2431-10-25 20416046 PMC2873518

[79] Schoemaker AA Sprikkelman AB Grimshaw KE Roberts G Grabenhenrich L Rosenfeld L et al. Incidence and natural history of challenge-proven cow’s milk allergy in European children–EuroPrevall birth cohort. *Allergy*. (2015) 70:963–72. 10.1111/all.12630 25864712

[80] Flom JD Sicherer SH. Epidemiology of cow’s milk allergy. *Nutrients*. (2019) 11:1051. 10.3390/nu11051051 31083388 PMC6566637

[81] Cruchet S Arancibia ME Maturana A Marchant P Rodríguez L Lucero Y. Prevalence of cow’s milk allergy in infants from an urban, low-income population in Chile: a cohort study. *Nutrients*. (2025) 17:1859. 10.3390/nu17111859 40507128 PMC12157014

[82] Arancibia ME Lucero Y Miquel I Marchant P Rodriguez L Alliende F et al. Association of cow’s milk protein allergy prevalence with socioeconomic status in a cohort of Chilean infants. *J Pediatr Gastroenterol Nutr*. (2020) 71:e80–3. 10.1097/MPG.0000000000002787 32427653

[83] Mehaudy R Parisi C Petriz N Eymann A Jáuregui M Orsi M. Prevalence of cow’s milk protein allergy among children in a university community hospital. *Archivos argentinos de pediatria.* (2018) 116:219–23. 10.5546/aap.2018.eng.219 29756713

[84] El-Sayed ZA El-Owaidy RH Abd El-Lateef HM Hammouda AS. Screening for cow milk allergy among young Egyptian children. *QJM Int J Med*. (2021) 114:hcab113.004. 10.1093/qjmed/hcab113.004

[85] de Oliveira LCL Silva LR Franco JM Watanabe AS Pinto Júnior AB Capelo A et al. Atualização em Alergia Alimentar 2025: posicionamento conjunto da Associação Brasileira de Alergia e Imunologia e Sociedade Brasileira de Pediatria. *Arquivos de Asmas Alergia e Imunologia*. (2025) 9:5–96. 10.5935/2526-5393.20250003

[86] Labrosse R Graham F Caubet JC. Non-IgE-mediated gastrointestinal food allergies in children: an update. *Nutrients*. (2020) 12:2086. 10.3390/nu12072086 32674427 PMC7400851

[87] Lapa Filho C Lapa H Franco J Vieira S Solé D Vieira M et al. Cow’s milk protein allergy and primary health care: a narrative review of current guidelines. *Residência Pediátrica.* (2022) 12:1–9. 10.25060/residpediatr-2022.v12n3-526

[88] Gonçalves LC Guimarães TC Silva RM Cheik MF de Ramos Nápolis AC Barbosa ESG et al. Prevalence of food allergy in infants and pre-schoolers in Brazil. *Allergol Immunopathol (Madr)*. (2016) 44:497–503. 10.1016/j.aller.2016.04.009 27496782

[89] Al-Beltagi M Saeed NK Bediwy AS Bediwy HA Elbeltagi R. Cow milk protein allergy mimics in infancy. *World J Clin Pediatr*. (2025) 14:103788. 10.5409/wjcp.v14.i3.103788 40881085 PMC12305122

[90] Facioni MS Raspini B Pivari F Dogliotti E Cena H. Nutritional management of lactose intolerance: the importance of diet and food labelling. *J Transl Med*. (2020) 18:260. 10.1186/s12967-020-02429-2 32590986 PMC7318541

[91] Misselwitz B Butter M Verbeke K Fox MR. Update on lactose malabsorption and intolerance: pathogenesis, diagnosis and clinical management. *Gut*. (2019) 68:2080–91. 10.1136/gutjnl-2019-318404 PMC6839734 31427404

[92] EFSA Panel on Dietetic Products, Nutrition and Allergies (NDA). Scientific opinion on lactose thresholds in lactose intolerance and galactosaemia. *EFSA J.* (2010) 8:1777. 10.2903/j.efsa.2010.1777

[93] Sharp E D’Cunha NM Ranadheera CS Vasiljevic T Panagiotakos DB Naumovski N. Effects of lactose-free and low-lactose dairy on symptoms of gastrointestinal health: a systematic review. *Int Dairy J*. (2021) 114:104936. 10.1016/j.idairyj.2020.104936

[94] Simren M Palsson OS Whitehead WE. Update on Rome IV criteria for colorectal disorders: implications for clinical practice. *Curr Gastroenterol Rep*. (2017) 19:15. 10.1007/s11894-017-0554-0 28374308 PMC5378729

[95] Marx M Maye H Abdelrahman K Hessler R Moschouri E Aslan N et al. Maladies fonctionnelles digestives: mise au point concernant la classification Rome IV. *Revue Médicale Suisse*. (2018) 14:1512–6. 10.53738/revmed.2018.14.616.1512 30156785

[96] Bertin L Zanconato M Crepaldi M Marasco G Cremon C Barbara G et al. The role of the FODMAP diet in IBS. *Nutrients*. (2024) 16:370. 10.3390/nu16030370 38337655 PMC10857121

[97] Alrasheedi AA Jahlan EA Bakarman MA. The effect of low-FODMAP diet on patients with irritable bowel syndrome. *Sci Rep*. (2025) 15:16382. 10.1038/s41598-025-01163-3 40350523 PMC12066726

[98] Varjú P Gede N Szakács Z Hegyi P Cazacu IM Pécsi D et al. Lactose intolerance but not lactose maldigestion is more frequent in patients with irritable bowel syndrome than in healthy controls: a meta-analysis. *Neurogastroenterol Motil*. (2019) 31:e13527. 10.1111/nmo.13527 30560578 PMC7379306

[99] Meng Y Zhou Y Li H Chen Y Dominik G Dong J et al. Effectiveness of growing-up milk containing only A2 β-casein on digestive comfort in toddlers: a randomized controlled trial in China. *Nutrients*. (2023) 15:1313. 10.3390/nu15061313 36986042 PMC10058316

[100] Sheng X Li Z Ni J Yelland G. Effects of conventional milk versus milk containing only A2 β-casein on digestion in Chinese children: a randomized study. *J Pediatr Gastroenterol Nutr*. (2019) 69:375–82. 10.1097/MPG.0000000000002437 31305326 PMC6727941

[101] Malagelada JR Accarino A Azpiroz F. Bloating and abdominal distension: old misconceptions and current knowledge. *Am J Gastroenterol*. (2017) 112:1221–31. 10.1038/ajg.2017.129 28508867

[102] Mari A Abu Backer F Mahamid M Amara H Carter D Boltin D et al. Bloating and abdominal distension: clinical approach and management. *Adv Ther*. (2019) 36:1075–84. 10.1007/s12325-019-00924-7 30879252 PMC6824367

[103] Bartley J McGlashan SR. Does milk increase mucus production? *Med Hypotheses*. (2010) 74:732–4. 10.1016/j.mehy.2009.10.044 19932941

[104] Wüthrich B Schmid A Walther B Sieber R. Milk consumption does not lead to mucus production or occurrence of asthma. *J Am Coll Nutr*. (2005) 24:547S–55S. 10.1080/07315724.2005.10719503 16373954

[105] Koren Y Armoni Domany K Gut G Hadanny A Benor S Tavor O et al. Respiratory effects of acute milk consumption among asthmatic and non-asthmatic children: a randomized controlled study. *BMC Pediatr*. (2020) 20:433. 10.1186/s12887-020-02319-y 32919454 PMC7488715

[106] Koletzko B Demmelmair H Grote V Prell C Weber M. High protein intake in young children and increased weight gain and obesity risk. *Am J Clin Nutr*. (2016) 103:303–4. 10.3945/ajcn.115.128009 26791192

[107] Koletzko BV Luque V Grote V Totzauer M. Is growth in early childhood a window of opportunity for programming long-term health? *Ann Nutr Metab*. (2024) 80:29–38. 10.1159/000545315 40414207

[108] Institute of Medicine. *Dietary Reference Intakes for Energy, Carbohydrate, Fiber, Fat, Fatty Acids, Cholesterol, Protein, and Amino Acids.* Washington (DC): National Academies Press (2005).

[109] Agostoni C Turck D. Is cow’s milk harmful to a child’s health? *J Pediatr Gastroenterol Nutr*. (2011) 53:594–600. 10.1097/MPG.0b013e318235b23e 21921812

[110] Matthews JJ Arentson-Lantz EJ Moughan PJ Wolfe RR Ferrando AA Church DD. Understanding dietary protein quality: digestible indispensable amino acid scores and beyond. *J Nutr*. (2025) 155:3152–67. 10.1016/j.tjnut.2025.07.005 40675340 PMC12799415

[111] Cuenca-Sánchez M Navas-Carrillo D Orenes-Piñero E. Controversies surrounding high-protein diet intake: satiating effect and kidney and bone health. *Adv Nutr*. (2015) 6:260–6. 10.3945/an.114.007716 25979491 PMC4424780

[112] FAO and WHO. *Sustainable Healthy Diets – Guiding Principles.* Rome: FAO and WHO (2019).

[113] Peterson CB Mitloehner FM. Sustainability of the dairy industry: emissions and mitigation opportunities. *Front Animal Sci*. (2021) 2:760310. 10.3389/fanim.2021.760310

[114] Priyashantha H. World dairy system sustainability: a milk quality perspective. *Front Sustain Res Manag*. (2025) 4:1572962. 10.3389/fsrma.2025.1572962

[115] FAO, IFAD, UNICEF, WFP, WHO. *The State of Food Security and Nutrition in the World 2024: Financing to End Hunger, Food Insecurity and Malnutrition in All Its Forms.* Rome: FAO (2024).

[116] Herber C Bogler L Subramanian SV Vollmer S. Association between milk consumption and child growth for children aged 6-59 months. *Sci Rep*. (2020) 10:6730. 10.1038/s41598-020-63647-8 32317668 PMC7174323

[117] Hidayat K Zhang LL Rizzoli R Guo YX Zhou Y Shi YJ et al. The effects of dairy product supplementation on bone health indices in children aged 3 to 18 years: a meta-analysis of randomized controlled trials. *Adv Nutr*. (2023) 14:1187–96. 10.1016/j.advnut.2023.06.010 37414219 PMC10509403

[118] de Lamas C de Castro MJ Gil-Campos M Gil Á Couce ML Leis R. Effects of dairy product consumption on height and bone mineral content in children: a systematic review of controlled trials. *Adv Nutr*. (2019) 10:S88–96. 10.1093/advances/nmy096 31089738 PMC6518138

[119] Rizzoli R. Dairy products and bone health. *Aging Clin Exp Res*. (2022) 34:9–24. 10.1007/s40520-021-01970-4 34494238 PMC8794967

[120] Zhao ZF Li BY He Q Hao JY Zhang KS Zhang B et al. Impact of dairy supplementation on bone acquisition in children’s limbs: a 12-month cluster-randomized controlled trial and meta-analysis. *Arch Osteoporos*. (2024) 19:65. 10.1007/s11657-024-01422-2 39043915

